# Biomonitoring of Heavy Metal Toxicity in Freshwater Canals in Egypt Using Creeping Water Bugs (*Ilyocoris cimicoides*): Oxidative Stress, Histopathological, and Ultrastructural Investigations

**DOI:** 10.3390/antiox13091039

**Published:** 2024-08-27

**Authors:** Lamia M. El-Samad, Esraa A. Arafat, Ola Mohamed Nour, Nessrin Kheirallah, Mohammed E. Gad, Mohamed Hagar, Zeinab A. El-Moaty, Mohamed A. Hassan

**Affiliations:** 1 Department of Zoology, Faculty of Science, Alexandria University, Alexandria 21321, Egypt; lamya.moustafa@alexu.edu.eg (L.M.E.-S.); esraa.arafat@alexu.edu.eg (E.A.A.); nessrin.kheirallah@alexu.edu.eg (N.K.); 2 Department of Biological and Geological Sciences, Faculty of Education, Alexandria University, Alexandria 21526, Egypt; onour@alexu.edu.eg; 3 Department of Zoology and Entomology, Faculty of Science, Al-Azhar University, Nasr City, Cairo 11884, Egypt; mohamed.gad9@azhar.edu.eg; 4 Department of Chemistry, Faculty of Science, Alexandria University, Alexandria 21321, Egypt; mohamed.hagar@alexu.edu.eg; 5 Department of Biological Sciences, College of Science, King Faisal University, Al-Ahsa 31982, Saudi Arabia; 6 Protein Research Department, Genetic Engineering and Biotechnology Research Institute (GEBRI), City of Scientific Research and Technological Applications (SRTA-City), New Borg El-Arab City, Alexandria 21934, Egypt

**Keywords:** antioxidant parameters, aquatic bug, freshwater pollution, free radicals, heavy metals, *Ilyocoris cimicoides*, midgut tissues, Malpighian tubules, oxidative stress, ovarioles

## Abstract

The abundance of metal pollutants in freshwater habitats poses serious threats to the survival and biodiversity of aquatic organisms and human beings. This study intends for the first time to assess the pernicious influences of heavy metals in Al Marioteya canal freshwater in Egypt, compared to Al Mansoureya canal as a reference site utilizing the creeping water bug (*Ilyocoris cimicoides*) as an ecotoxicological model. The elemental analysis of the water showed a significantly higher incidence of heavy metals, including cadmium (Cd), cobalt (Co), chromium (Cr), nickel (Ni), and lead (Pb), in addition to the calcium (Ca) element than the World Health Organization’s (WHO) permitted levels. The Ca element was measured in the water samples to determine whether exposure to heavy metals-induced oxidative stress engendered Ca deregulation in the midgut tissues of the creeping water bug. Remarkably, increased levels of these heavy metals were linked to an increase in chemical oxygen demand (COD) at the polluted site. Notably, the accumulation of these heavy metals in the midgut tissues resulted in a substantial reduction in antioxidant parameters, including superoxide dismutase (SOD), catalase (CAT), glutathione peroxidase (GPx), and ascorbate peroxidase (APOX), along with a marked rise in malondialdehyde (MDA), cytochrome P450, and protein carbonyl levels. These results clearly indicate a noticeable disturbance in the antioxidant defense system due to uncontrollable reactive oxygen species (ROS). Notably, the results demonstrated that oxidative stress caused disturbances in Ca levels in the midgut tissue of *I. cimicoides* from polluted sites. Furthermore, the comet and flow cytometry analyses showed considerable proliferations of comet cells and apoptotic cells in midgut tissues, respectively, exhibiting prominent correlations, with pathophysiological deregulation. Interestingly, histopathological and ultrastructural examinations exposed noticeable anomalies in the midgut, Malpighian tubules, and ovarioles of *I. cimicoides*, emphasizing our findings. Overall, our findings emphasize the potential use of *I. cimicoides* as a bioindicator of heavy metal pollution in freshwater to improve sustainable water management in Egypt.

## 1. Introduction

In a rapidly globalized world, many risks have arisen. One such risk is water pollution, which is rising nowadays as an international environmental burden with the expansion of urbanization, increasing industrialization, climate change, and anthropogenic activities [1,2,3]. The risk posed by water pollution is elevated in developing countries, particularly in their rural and agricultural communities, where they are mainly depending on natural resources and a healthy ecosystem for their livelihoods [4,5]. In Egypt, the main source for drinking and irrigation is the Nile River and its network of streams and canals [6]. Nevertheless, the Egyptian River Nile is overwhelmed with continuous multiple environmental pressures, including erosion, degradation, agriculture drainage, and the discharging of untreated water wastes and hazardous chemicals and materials [7,8].

The Al Marioteya canal contains freshwater that branches off from the Nile River in the Beni Suef Governorate (south of Cairo) and pours into El-Qanater in Qalyubia Governorate (north of Cairo). Its bank is mainly used for domestic purposes, irrigation, and fishing, but it suffers critically from the continuous dumping of waste into it. Thus, it is well known as one of the heavily contaminated streams along the Nile, being mainly polluted with agricultural drainage and industrial effluents, with high levels of phenol and polycyclic aromatic hydrocarbons [9,10]. Among environmental stresses, metal pollution is considered a serious and prominent pollutant in aquatic ecosystems [8,11]. Although many elements are essential in small quantities, concentrations above a certain threshold can be toxic to diverse organisms [12,13]. Heavy metals occur naturally in aquatic ecosystems; however, the natural flux of these metals has increased over the last few decades, posing a threat to aquatic ecosystems [8,14]. Sources of heavy metal input, either in particulate or dissolved form, include antifouling boat paints, urban storm water run-off, mining and industrial effluents, and atmospheric depositions [15]. Heavy metals are often persistent, non-biodegradable, and have the tendency to accumulate in aquatic organisms, either directly (by accumulating in their bodies) or indirectly (through food) [16,17]. Therefore, human exposure to heavy metals originated from aquatic source is inevitable. It is assumed that heavy metals often interact with living organisms by undergoing oxidation, losing electrons, and generating metal cations that have a strong affinity to the nucleophilic sites of key macromolecules [18]. Thus, various acute and chronic detrimental consequences of heavy metals impact different organs in the body. Consequences emerging from the detrimental impact of heavy metals include gastrointestinal and kidney malfunction, neurological diseases, skin lesions, vascular impairment, immunological disorders, birth defects, and cancer [18,19,20,21,22]. Critically, exposure to multiple metals at the same time could incite cumulative pernicious influences [18,20].

A previous study demonstrated that the amounts of iron (Fe), lead (Pb), and manganese (Mn) in the water samples collected from three locations along the Nile River in Egypt were higher than the safe limits set by the World Health Organization (WHO). On the other hand, copper (Cu) and zinc (Zn) were detected to be within the allowed limits [23]. The bioaccumulation process of heavy metals entails the exploitation of some aquatic species as useful bioindicators for aquatic environments to evaluate the health status of ecosystems [17,24,25].

In general, insects are considerably diverse and widely distributed, mostly in all habitats and at all trophic levels [26,27,28]. In addition to their minimal ethical problems, insects are broadly utilized as a good bioindicator of environmental stress [26,27,28]. In freshwater ecosystems, aquatic insects are the dominant taxon group [25,27] and are considered to be sentinels in lotic ecosystems [29]. Previous studies have demonstrated the eminent sensitivity of aquatic insects as indicators of environmental changes in water and habitat quality, leading to their frequent use as bioindicators in heavy metal pollution biomonitoring programs [27,29]. Aquatic Hemipterans, a type of insect, are typically endemic to specific regions and islands, often exhibiting a restricted distribution, which serves as a bioindicator of their habitat [30]. About 5000 species of true bugs (Heteropterans) inhabit aquatic and semi-aquatic habitats worldwide, making them the most diverse group of hemimetabolous insects in freshwater ecosystems [31]. Globally, the infraorder Nepomorpha comprises 11 families and 2309 species in freshwater biotopes, of which approximately 230 species are known from the Palearctic region [31,32].

Beside their remarkable economic role in mosquito control, Nepomorphan species are known as food sources for many vertebrate species and are widely used in both fish and poultry feeding [33,34]. Nevertheless, recent studies have only focused on their sensitivity to pesticides and oil spill pollution, despite their great value as bioindicators for monitoring pollution [34,35]. For instance, the creeping water bug, *Ilyocoris cimicoides* (infraorder: Nepomorpha, family: Naucoridae), exhibits high resistance to fluctuations in abiotic factors, despite the fact that species usually found within the same habitat are sensitive to pesticides and insecticides [32,36,37]. In this context, awareness of using the appropriate biomarkers that provide early warning signals to stress caused by heavy metal toxicity is critical for ecological relevance and is increasingly being advocated [38]. In risk assessment frameworks such as the European Union Water Framework Directive and the Egyptian Water Policy, the analysis of organisms’ biomarker responses is crucial for policies and management strategies [38,39].

Under pollution stress, massive amounts of heavy metals accumulate in the tissues of organisms, inciting the overproduction of reactive oxygen species (ROS) [40,41]. The increase in ROS generation may affect cellular integrity and impair cellular mechanisms through lipid peroxidation, one of the most frequent cellular injury processes where ROS react with membrane-associated lipids [40,41,42,43]. It is believed that ROS production is controlled by efficient antioxidant capacity, characterized by a set of antioxidant enzymes that function together to detoxify ROS [40,41,42,43]. Antioxidant enzymes, including superoxide dismutase (SOD), catalase (CAT), ascorbate peroxidase (APOX), and glutathione peroxidase (GPx), have shown their effective function as biomarkers for pollution in many organisms [40,41,44]. Likewise, genetic damage is one of the most useful tools for monitoring environmental risks [44,45].

In light of the aforementioned reasons and for improving the environmental quality and sustainable development monitoring in the Egyptian ecosystem, the current study seeks to investigate heavy metal accumulation along the Egyptian Nile River, Giza Governorate, from both less and highly polluted areas (the Al Mansoureya and the Al Marioteya canals, respectively) utilizing the creeping water bug *I. cimicoides* as a monitoring organism. Although previous studies probed the response of some aquatic bugs to different pollutants using biochemical markers, few studies were carried out on water bugs following histopathological examinations to demonstrate the alterations in different organs obtained from clean and polluted water [34]. Moreover, no previous investigations inspected different organs from control and polluted aquatic bugs, including the midgut, Malpighian tubules, and ovaries, as extensively illustrated in our current study. In order to comprehend the stress response of heavy metals pollution, we used a multi-biomarker approach and tissue features by investigating the following parameters in insect tissues: (1) metal concentration using energy-dispersive X-ray microanalysis (EDX); (2) levels of cytochrome P450, protein carbonyl, and lipid peroxidation, in addition to the activity of antioxidant enzymes (SOD, CAT, GPx, and APOX); (3) genotoxicity to determine DNA impairment and cellular apoptosis using comet and annexin assays, respectively; and (4) histological and ultrastructure malformations in the midgut, Malpighian tubules, and ovaries of *I. cimicoides*.

## 2. Materials and Methods

### 2.1. Study Area

**Figure 1:**
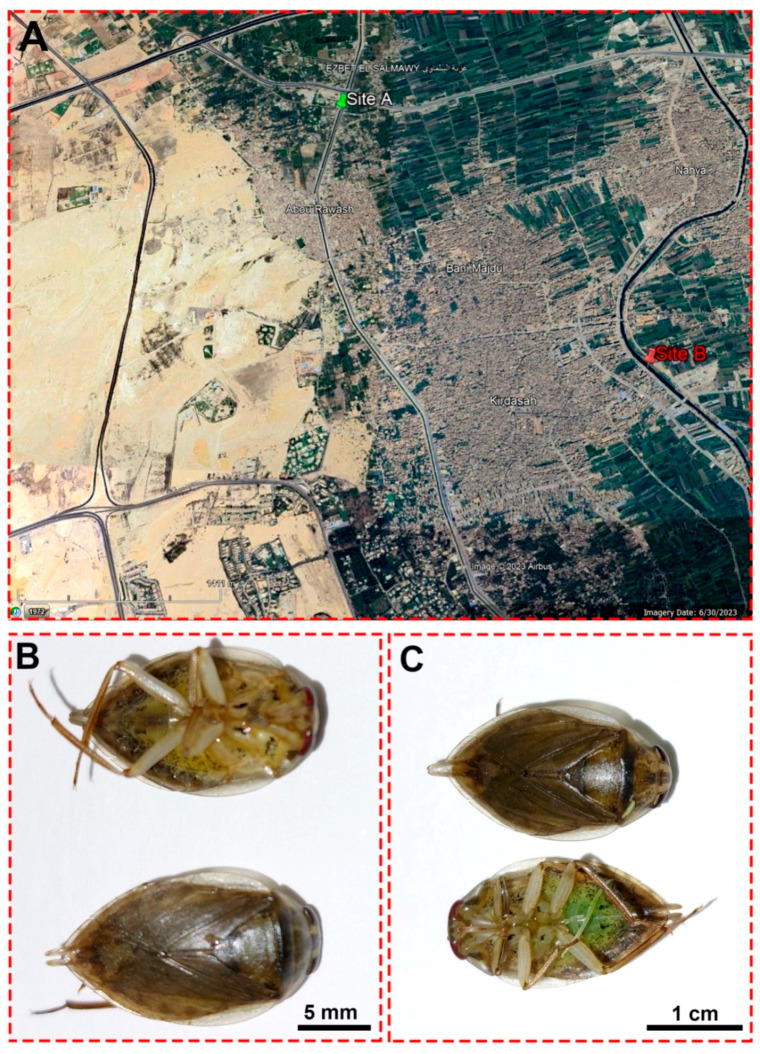
(**A**) A satellite image from Google Earth shows the locations of the Al Mansoureya (site A) and Al Marioteya (site B) canals. (**B**) An adult male *Ilyocoris cimicoides* and (**C**) adult female *Ilyocoris cimicoides* displaying their morphological features.

Water and insect samples were collected on 13 March 2022 from two sites: the Al Mansoureya and Al Marioteya canals, designated as sites A (southern Egypt, longitude: 31°5′45.67″ E; latitude: 30°3′11.43″ N) and B (southern Egypt, longitude: 31°7′18.30″ E; latitude: 30°1′59.42″ N), respectively, as depicted in Figure 1. The Al Mansoureya and Al Marioteya canals run through Giza City and are located in the Giza Governorate, southern Egypt, and the distance between both collection sites is about 4.1 km. In this study, we designated site A as a reference or non-polluted site, whereas we assigned site B as a polluted site because of waste disposal in the Al Marioteya freshwater canal, which adversely impacts the organisms and plants. As a result, we compared the water quality and the accumulation of heavy metals in the midgut tissues of the studied aquatic bug at both sites.

### 2.2. Water Sampling and Analysis

At both sampling sites, three surface water samples were randomly collected on 13 March 2022 and transported to the laboratory in polythene bottles under refrigeration. Temperature and pH were measured in the field employing a portable instrument (WTW Cond 3110 equipped with a Tetracon 325 electrode (Xylem Analytics Germany, Weilheim, Germany) and the WTW 3310 pH-meter (Xylem Analytics Germany, Weilheim, Germany), equipped with a SenTix 81 pH electrode, respectively).

#### 2.2.1. Total Nitrogen Content

To estimate the total nitrogen content in water samples, 75 mL of the sample was digested on a Dk-20 heating digester (Velp Scientifica, Usmate Velate, Italy), with H_2_SO_4_, K_2_SO_4,_ and CuSO_4_, at 400 °C. After cooling, the samples were distilled using a Velp Scientifica distillation system by automatically adding 10 mL of 10% NaOH, and the distilling solution was ammonia-free water. The captured solution was 0.25 N HCl (standardized by Na_2_CO_3_), which was back-titrated vs. 0.5 N NaOH.

#### 2.2.2. Chemical Oxygen Demand (COD), pH, and Nutritional Salts Measurements

pH, total dissolved salts (TDS), and nutritional salts such as phosphate, ammonia, nitrate, and nitrite (PO4-P, NH4-N) were analyzed using the American Public Health Association standard procedures for the examination of natural and treated wastewater (APHA 1975) [46]. The chemical oxygen demand (COD) was assessed using a colorimetric approach, which relies on the oxidation of the reducing materials in the water sample by a potassium dichromate solution (K_2_Cr_2_O_7_) in an acidic solution, following the previously reported approach [47]. Thus, 50 mL of water samples collected for the two sites were put in a reflux flask before being thoroughly mixed with 10 mL of K_2_Cr_2_O_7_ solution comprising 1 g of mercuric sulfate. Beads with anti-bumping properties were utilized to govern the boiling process of the solution. Subsequently, 10 mL of concentrated sulfuric acid comprising silver sulfate was carefully added into the condenser through its open end. The mixture was then thoroughly blended by employing a swirling motion. The reflux apparatus was adjusted for 1 h and then let to cool at room temperature. Afterward, the flask was taken out, and its contents were diluted to a volume of 150 mL using distilled water. Following this, three drops of the ferroin indicator were added to the resultant solution. The provided sample underwent titration using ferrous ammonium sulfate until reaching the endpoint, at which the blue-green color transitioned to reddish-brown. The COD of the blank sample was subsequently computed. The measurements were conducted by means of the Benchtop spectrophotometer (Model DR 3900 HACH, Loveland, CO, USA).

#### 2.2.3. Heavy Metals Determination in Water Samples

The concentrations of heavy metals, including cobalt (Co), nickel (Ni), chromium (Cr), cadmium (Cd), and lead (Pb), along with the calcium (Ca) element in water samples were determined by means of atomic absorption (Analytik Jena CONTRAA 330; Jena, Germany), following the recommended conditions and detection limits (DL) in the manual for each metal.

### 2.3. Collection of Aquatic Bug (Ilyocoris cimicoides)

Water bugs were captured from the two study sites on 13 March 2022, using a 2 mm mesh pond net (25 × 25 cm head), which was dipped into the water surface. All collected organisms associated with marsh plants were transported immediately to the laboratory of the Zoology Department, Faculty of Science, Alexandria University, Egypt, in plastic aquaria (40 × 26 × 24 cm) filled with 3 l of aerated water at 18 ± 1 °C, within a temperature-constant room, under a LED light, and during a photoperiod of 12:12 h. The aquatic bug was identified as *Ilyocoris cimicoides*, and 180 individuals (8–15 mm body length) from each region were randomly utilized in this study. The aquatic bugs were dissected on ice within the day of collection under a dissecting stereomicroscope (Leica M205C, Heerbrugg, Switzerland), and the majority of the insects were characterized as females, as shown in Figure 1B,C. Therefore, we performed the investigations using the different organs harvested from female *I. cimicoides*. For biochemical evaluations, midgut tissues were dissected and directly preserved at −80 °C for the corresponding investigations. On the other hand, the rest of the aquatic bugs were injected with 0.02 mL of 4% formaldehyde:1% glutaraldehyde (4F:1G) buffer (pH 7.2) before being anatomized following the previously described method [48] to utilize the midgut, Malpighian tubules, and ovaries for inspecting their histological and ultrastructure attributes.

### 2.4. Determination of Heavy Metals in Aquatic Bug

Five individuals of adult females *I. cimicoides* (three sections from each creeping water bug) obtained from each study site were utilized to measure the heavy metals concentration, including Co, Ni, Cr, Cd, and Pb, in addition to the Ca element in the midguts, by means of energy-dispersive X-ray analysis (EDX) connected to a scanning electron microscope (Jeol JSM-5300, Tokyo, Japan). Using SEM-EDX software, different peaks were characterized by measuring line intensity for each element in the sample and comparing it with the reference element.

### 2.5. Biochemical Determinations in the Midgut of I. cimicoides

To estimate the oxidative stress biomarkers in *I. cimicoides*, midgut tissues from the two sites were weighted and homogenized in a phosphate buffer (pH 7.0) for 30 s, before being clarified for 30 min at 5000× *g* and 4 °C, and then stored at −80 °C until analysis. As a marker of lipid peroxidation, malondialdehyde (MDA) concentration was determined following the previous method [49], while protein carbonyl was estimated using a respective kit (Cat. No. 10005020, Cayman Chemical, Solana Beach, CA, USA). The activities of catalase (CAT) and glutathione peroxidase (GPx) were evaluated following the methods of Aebi [50] and Chu et al. (1993) [51], respectively. Additionally, the superoxide dismutase (SOD) activity was assessed by the ESOD-100 kit (Cat. No. ESOD-100, BioAssay Systems, Hayward, CA, USA), while the ascorbate peroxidase (APOX) activity was measured using a corresponding kit (Cat. No. CAK1052, Cohesion Biosciences, London, UK).

Furthermore, the cytochrome P450 activity was assessed in the homogenates of the midgut tissues in accordance with previously reported procedures [52], with minor adjustments. Thus, 20 mg of tetramethylbenzidine (TMBZ) was dissolved in 25 mL methanol, followed by the addition of 75 mL of 0.25 M sodium acetate buffer (NaOAc, pH 5.0). The positive control was prepared by dissolving 10 mg of cytochrome-C in 100 mL of 0.25 M NaOAc buffer (pH 5.0). The wells representing the positive control were loaded with 100 μL of cytochrome-C, while 100 μL was loaded from the insect homogenates into the respective wells of the microplate, followed by the pipetting of 100 μL of potassium phosphate in all wells. Afterward, 200 μL of TMBZ solution was loaded into each test well. To commence the reaction, 25 μL of 3% hydrogen peroxide (H_2_O_2_) was added to each well before being incubated at room temperature for 5 min, and the optical densities were then estimated at 620 nm by means of a microplate reader to evaluate the oxidase activity. The activity of cytochrome P450 was calculated in relation to the protein content accordingly. All biochemical measurements were conducted in five replicates.

### 2.6. DNA Impairment Assay

For assessing the impaired DNA in *I. cimicoides*, a comet assay was used following the previously published protocol [42,53]. Following the dissection, the midgut was isolated and sliced into small pieces, followed by homogenization in a clod buffer (0.075 M NaCl and 0.024 M Na_2_EDTA), and then clarified for 10 min at 700× *g* and 4 °C. After washing the cell pellet thrice with the similar buffer, the cell suspension was blended with low-melting-point agarose (1:9, *v*/*v*) before being instantly spread onto frosted microscope slides, which were overlaid with normal-melting-point agarose. Following this, the slides were submerged in lysis buffer (2.5 M NaCl, 100 mM EDTA, 10 mM Tris, 1% Triton X-100, 10% DMSO, pH 10.0) for 24 h and then underwent electrophoresis in a buffer (1 mM EDTA, 300 mM NaOH, pH 13.0) at 300 mA and 4 °C for 20 min. After the neutralization of the slides for 15 min in the buffer, the slides were dried before being stained with ethidium bromide (20 mg/mL). We visualized the comet assay using a fluorescent microscope (LEICA DMLS, ×400 magnification, Olympus, Tokyo, Japan) attached to a U-MNG filter. We investigated three slides, examining about 100 randomly selected cells per slide to ascertain tail length, percentage of tail DNA, and tail moment.

### 2.7. Flow Cytometry Assay for Detection of Apoptotic and Necrotic Cells

To probe apoptotic and necrotic cells in the midgut tissue of *I. cimicoides*, the TACS Annexin V-FITC apoptosis kit (Cat. No. TA4638, Sigma Aldrich, Darmstadt, Germany) was employed according to the procedures supplied by the manufacturer. Midgut isolated from aquatic bugs from both sites was homogenized in a cold phosphate buffer saline (PBS, pH 7.4) at 4 °C. The cells were harvested, washed thrice in PBS, resuspended in a volume of 195 μL of binding buffer, and then 5 μL of Annexin-V-FITC reagent was added to the cell suspension. After 15 min of incubation in the dark, the cells were collected and mixed again in 190 μL of binding buffer. Afterward, 10 μL of propidium iodide solution was then added. The specimens were examined by means of flow cytometry (Becton Dickinson, Franklin Lakes, NJ, USA) and the data were analyzed employing Cell Quest Pro software version 5.2.1, 2005 (Becton Dickinson, San Jose, CA, USA).

### 2.8. Histological and Ultrastructure Investigations

Immediately after dissection, the midgut, Malpighian tubules, and ovaries of *I. cimicoides* (5 individuals/site) were fixed in the 4F:1G solution that was prepared in 0.1 M phosphate buffer solution (pH 7.2) at 4 °C for 3 h before being postfixed in 2% OsO_4_ solution at 4 °C for 2 h. Afterward, the tissues were dehydrated in a series of grades of ethanol (10 min/concentration). The tissues were then embedded in an Epon–Araldite mixture and cut into 0.5 µm slices using an LKB ultramicrotome (LKB Bromma 2088 Ultrotome, Leica Instruments, Bannockburn, IL, USA) for semithin analysis. Toluidine blue was then applied to label the tissues, and they were surveyed under a light microscope (Olympus CX31, Tokyo, Japan) [44]. After selecting the areas of interest, ultrathin sections (60 nm thick) were prepared and the tissues were stained with uranyl acetate and lead citrate, followed by investigation by means of a transmission electron microscope (TEM, JEM-1400 Plus, Tokyo, Japan).

### 2.9. Statistical Analysis

All examinations were performed in 3–6 independent experiments, and the statistical analysis was carried out using SPSS (Version 25, IBM Software, Inc., Chicago, IL, USA) and GraphPad Prism (Version 8, GraphPad Software Inc., San Diego, CA, USA). Before commencing the analysis, the normal (Gaussian) distribution of the data was carried out following the Shapiro–Wilk test, and the Student’s *t*-test was used to evaluate the significant differences between insects collected from two sites in all the investigated parameters. All results are expressed as mean ± SD, and the significant difference was considered at *p* ≤ 0.05, while the high significant differences were determined at *p* ≤ 0.01, *p* ≤ 0.001, and *p* ≤ 0.0001.

## 3. Results

### 3.1. Environmental Parameters of Water

The physiochemical parameters of the water samples are presented in Table 1. The results revealed that the water temperature and the pH values were almost comparable between the two sites. Other water parameters, however, were higher at the polluted site compared to the control site, with the exception of dissolved oxygen (DO), which measured 6.8 mg/mL in the polluted area compared to 7.9 mg/mL in the control area.

### 3.2. Determination of Metals Concentration in Water and Midgut of I. cimicoides

The mean concentration values (mg/L) of the determined metals in water sampled from the control and polluted sites are shown in Table 2. The data demonstrate the determination of six heavy metals, namely Cd, Co, Cr, Ni, and Pb, in addition to Ca, in the water samples from both sites. For the six metals assessed, the concentrations were higher in the polluted site than the control one, with a significant difference between the two locations (*p* ≤ 0.05). More precisely, the metal concentrations in water showed a descending order of concentrations, as follows: Ca > Pb > Cr > Co > Cd = Ni, and Ca > Co > Pb > Cd > Cr > Ni in the control and polluted sites, respectively. Of particular interest, the average values of Ca, Pb, and Cr in the samples from the polluted site exceeded their average control values by ~2 times, while for Ni, Co, and Cd by ~1.25, 29, and 16 times, respectively.

Additionally, Table 3 illustrates the accumulated metal percentages in the midgut tissues of *I. cimicoides* tissues collected from the two sites by EDX analysis. Interestingly, none of the metals were detected in the control midgut tissues, except Ca (0.27 ± 0.1%). By contrast, the midgut tissues of *I. cimicoides* from polluted area revealed the aggregation of all metals reported in water. The concentration of Ca reported the highest accumulated level in the midgut tissues from the polluted site (3.93 ± 0.28%). In contrast to the value recorded in the water from both locations, Ni recorded the highest mean percentage value in the midgut tissue (0.73 ± 0.45%), followed by Co, Cd, and Pb, with mean percentage values of 0.54 ± 0.31%, 0.28 ± 0.37%, and 0.24 ± 0.22%, respectively, while Cr reported the lowest level in the tissue (0.07 ± 0.04%).

### 3.3. Oxidative Stress Biomarkers and Genotoxicity in Midgut Tissues of Creeping Water Bugs

**Figure 2:**
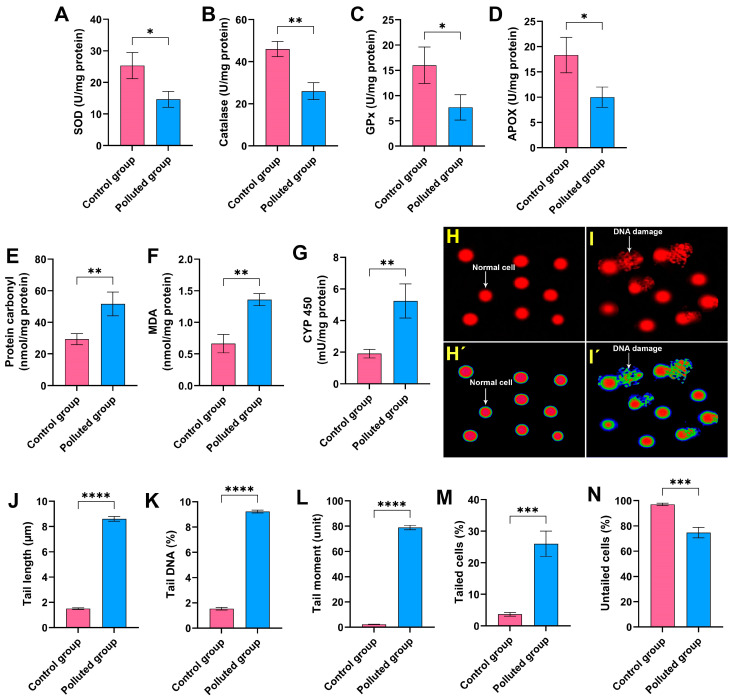
Assessment of (**A**) superoxide dismutase (SOD), (**B**) catalase, (**C**) glutathione peroxidase (GPx), (**D**) ascorbate peroxidase (APOX), (**E**) protein carbonyl, (**F**) malondialdehyde (MDA), and (**G**) cytochrome P450 (CYP 450) in the midgut homogenates of creeping water bugs from the polluted site compared to those from the clean site. (**H**,**I**) depict the image of the comet assay of cells from the midgut tissues of creeping water bugs from control and polluted sites, respectively, before being analyzed by ImageJ Fiji software (https://imagej.net/software/fiji/downloads) (accessed on 1 June 2024), as shown in (**H’**,**I’**). The comet evaluation results show (**J**) tail length, (**K**) DNA percentage in the comet tail, (**L**) tail moment, (**M**) tailed cells, and (**N**) untailed cells. All biochemical analyses were conducted from 3 to 6 replicates. To obtain the comet results, we randomly examined 100 comets per slide. Data were analyzed following the *t*-test, and all results are presented as mean ± SD. **** *p* < 0.0001, *** *p* < 0.001, ** *p* < 0.01, and * *p* < 0.05 indicate the significant differences between various parameters, which were estimated in the midgut tissues of creeping water bugs from site A versus site B.

The evaluation of oxidative stress biomarkers in the midgut homogenate of creeping water bug (*I. cimicoides*) collected from the polluted canal (site B) demonstrated substantial dysregulations compared to the aquatic bugs collected from the control site (site A), as delineated in Figure 2A–G. Remarkably, a significant diminution (*p* ≤ 0.05) in the antioxidant parameters responsible for the regulation of surplus ROS, including SOD, CAT, GPx, and APOX, was observed in the midgut tissues of the polluted group compared to those collected from the control site, as illustrated in Figure 2A–D. Moreover, it is discernible from the data in Figure 2E–G that the MDA, cytochrome P450, and protein carbonyl levels were markedly augmented in the polluted bug’s midgut tissues, with a significant difference (*p* ≤ 0.05) in relation to the control aquatic bugs. Overall, these results clearly suggest that the accumulation of heavy metals in the midgut tissues resulted in noticeable disorders in the oxidative stress biomarkers, implying the interference of the antioxidant defense system and redox homeostasis.

To evaluate the extent of DNA impairment in the midgut tissues provoked by the agglomeration of the six heavy metals, a comet assay was conducted. It is obvious from Figure 2H–N that the analysis of the comet assay results revealed a critical DNA impairment level in the midgut tissues obtained from the creeping bugs in the polluted site compared to those obtained from the clean site. In particular, the percentage of tailed cells in the polluted midgut tissues was 26 ± 4% compared to 3.7 ± 0.6% in the control midgut tissues, and the statistical analysis demonstrated significant differences between the two studied groups (*p* ≤ 0.05). Conversely, the rest of the examined cells were untailed, demonstrating a marked decrease in polluted midgut tissues compared to the control tissues. Other parameters, such as tail length, tail DNA, and the tail moment, which are related to the findings from the comet assay, showed notable malformation, with a particularly high ratio of tail DNA in the polluted tissues compared to the control tissues. Altogether, the comet assay results substantiate the deleterious genotoxic impact of the heavy metals in the Al Marioteya canal on the aquatic bug.

### 3.4. Flow Cytometry Assessment of Cell Viability in Creeping Water Bugs

**Figure 3:**
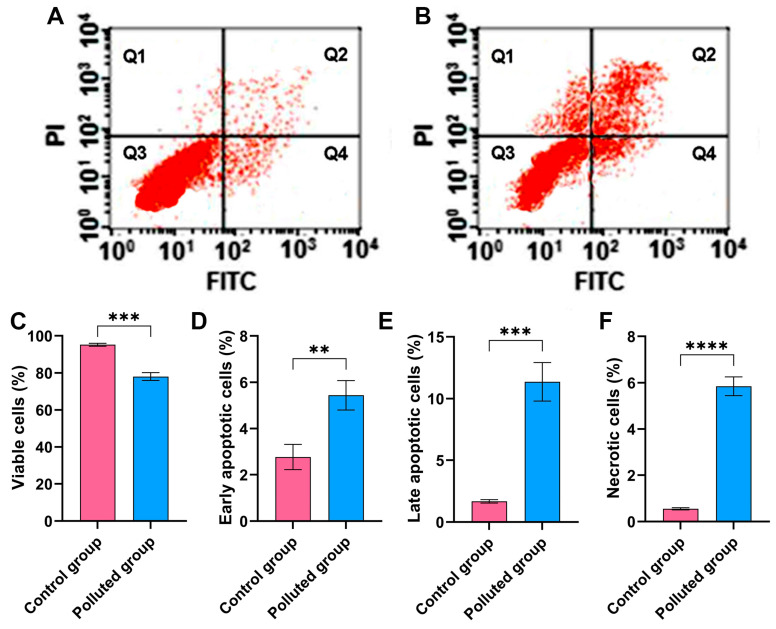
(**A**) Assessment of cell viability harvested from the midgut tissues of creeping water bugs collected from the control site and (**B**) creeping water bugs collected from the contaminated site. The data in (**A**,**B**), Q1, Q2, Q3, and Q4 point to necrotic, late apoptotic, viable, and early apoptotic cells, respectively. (**C**–**F**) depict the statistical analysis of flow cytometry results, demonstrating the difference between both sites in terms of viable, early apoptotic, late apoptotic, and necrotic cells. The flow cytometry analysis was carried out in three replicates. Data were analyzed following the *t*-test, and the values are shown as mean ± SD. **** *p* < 0.0001, *** *p* < 0.001, and ** *p* < 0.01 point to the significant differences among various parameters, which were estimated in the midgut tissues of creeping water bugs from site A versus site B.

The Annexin-V-FITC assay was performed to assess the impact of the six heavy metals detected in the contaminated water on the viability of cells harvested from the midgut tissues of creeping bugs, as depicted in Figure 3A,B. Critically, it is apparent from the quantification in Figure 3C that the cells from the polluted tissues manifested a substantial reduction in cell viability, with a value of 78.1 ± 2.2% compared to those from the control tissues, which showed a cell viability value of 95.2 ± 0.8%. Additionally, significant amplification in the percentage of necrotic, early apoptotic, and late apoptotic cells in the contaminated midgut could be recognized, compared to the healthy midgut tissues, as shown in Figure 3D–F. Notably, the statistical analysis emphasized significant differences between the polluted and control groups (*p* ≤ 0.05) in relation to all flow cytometeric determinants. Collectively, the heavy metals in the polluted water in the Al Marioteya canal (site B) elicited an extensive disturbance for creeping bugs cells, which corroborates the findings of oxidative stress and DNA impairment.

### 3.5. Histopathological and Ultrastructural Inspection of Midgut Tissues

**Figure 4:**
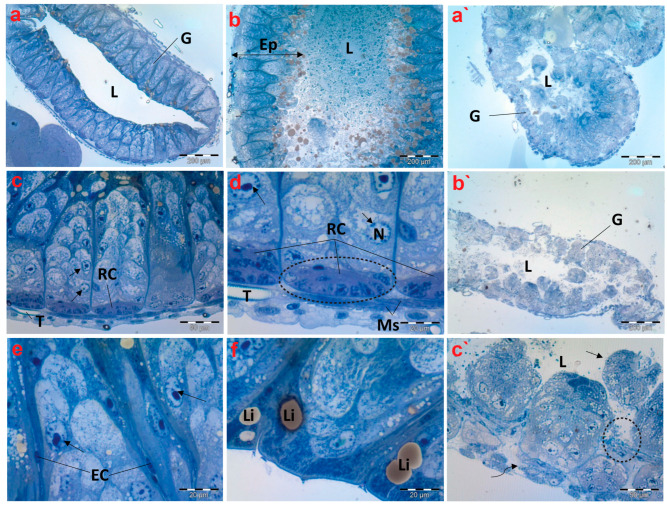
Photomicrographs of semithin sections exhibiting histological structures of the midgut of creeping water bugs collected from the reference site (A) and polluted site (B). (**a**–**f**) delineate the histological properties of the midgut of aquatic bugs from a clean site. (**a**) illustrates a transverse section through the midgut (G), and its Lumen (L). (**b**) shows the width of the midgut epithelium (Ep) and the contents within its lumen (L). (**c**,**d**) depict higher magnifications for the midgut, showing the muscle layer (Ms), regenerative cysts (RCs), and proliferative cells with normal nuclei. (arrows) nucleus (N), tracheole (T). (**e**) portrays active cell proliferation, proliferative cells (arrows), and endocrine cells (ECs). (**f**) exhibits lipid granules (Li) on the apical surface. (**a`**–**c`**) depict the histological anomalies in the midgut (G) of bugs from polluted water, demonstrating fragmentation of the midgut epithelium in the lumen (L) (arrow), lysis and separation of the muscle layer from the epithelium (curved arrow), and signs of epithelium lysis (encircled). (**a**) (scale bar = 200 µm and magnification ×100), (**b**) (scale bar = 200 µm and magnification ×100), (**c**) (scale bar = 50 µm and magnification ×400), (**d**) (scale bar = 20 µm and magnification ×1000), (**e**) (scale bar = 20 µm and magnification ×1000), (**f**) (scale bar = 20 µm and magnification ×1000), (**a`**) (scale bar = 200 µm and magnification ×100), (**b`**) (scale bar = 200 µm and magnification ×100), and (**c`**) (scale bar = 50 µm and magnification ×400).

In the control group, the histological studies of the midgut sections of creeping bugs revealed a typical structure, with digestive cells arranged in columns, regenerative crypts, and endocrine cells. Furthermore, the epithelial cell layer was well organized associated with a regular pattern of epithelial cells and normal contents within its lumen as displayed in Figure 4a,b. In addition, Figure 4c shows that a layer of circular striated muscles emerged to surround the epithelium of the midgut. Interestingly, the active proliferation and regeneration of cells were discerned. Additionally, typical regenerative cells were detectable in nests similar to those described by Fialho Mdo et al. [54], localized in the basal crypts of the midgut, along with differentiating or proliferative cells in the upper direction and typical tracheoles (Figure 4d). Figure 4e exhibits the scattered presence of rare endocrine cells throughout the epithelium. Additionally, many lipid droplets were observed beneath the apical membranes, as illustrated in Figure 4f.

Upon examining the histopathological characteristics of the midgut of creeping bugs from the contaminated area, the degeneration of midgut epithelium could be perceived, primarily provoked by a necrotic type of cell death. Most notably, histopathological attributes were recognized nearly in all the intestinal cells, as shown in Figure 4a`–c`. Precisely, severe structural impairment, combined with degeneration, was observed, including disorganized and disrupted midgut epithelium that lacked a characteristic morphology. Furthermore, the midgut epithelium separated from the underlying tissues, and some cells were discharged into the midgut’s lumen. In addition, many vacuoles were discernible inside the midgut epithelium. The endocrine cells were completely absent, and the muscle layer was aberrant and separated, compared to the control group.

**Figure 5:**
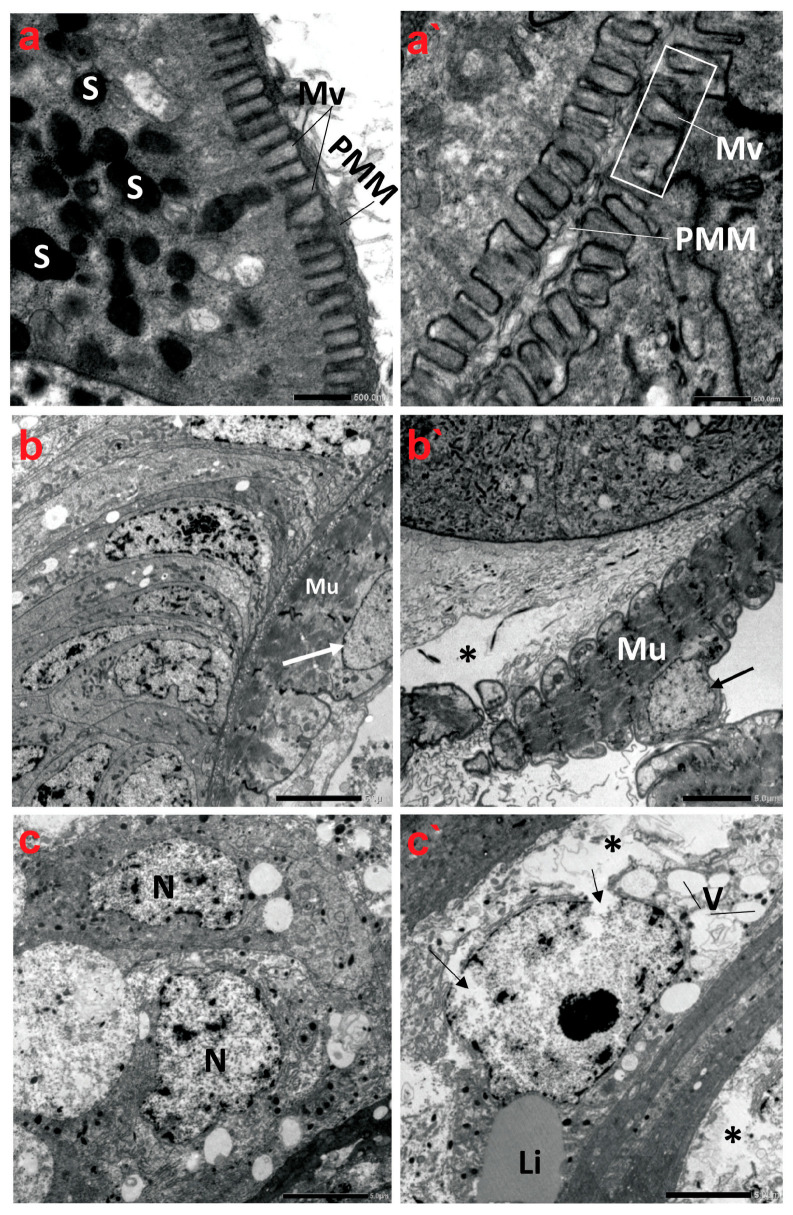
Transmission electron micrographs demonstrate the ultrastructure characteristics of the midgut of creeping water bugs collected from the reference site (A) and polluted site (B). (**a**–**c**) show the ultrastructure features of the midgut of aquatic bugs from a clean site. (**a**) displays the striated border of the midgut microvilli (Mv), perimicrovillar membrane (PMM), and secretory granules (S). (**b**) reveals normal muscle (Mu) morphology with a regular nuclear envelope (arrow). (**c**) illustrates the different shapes of the epithelium nuclei (N) with normal chromatin condensation. (**a`**–**c`**) exhibit ultrastructure aberrations in the midgut of bugs from polluted water, showing an irregularly striated border of the midgut. (**a`**) illustrates an irregularly structured brush border (microvilli, Mv; perimicrovillar membrane, PMM) and the absence of secretory granules. (**b`**) shows lysis of connective tissue (asterisk), abnormal muscle (Mu) morphology, and an irregular nuclear envelope (arrow), while (**c`**) exposes rupture of the nuclear envelope (arrows), lysis of the cytoplasm (asterisk), and vacuolation (V) with regular lipid droplet (Li). (**a**) (scale bar = 500 nm and magnification ×10K), (**b**) (scale bar = 5.0 µm and magnification ×1500), (**c**) (scale bar = 5.0 µm and magnification ×1500), (**a`**) (scale bar = 500 nm and magnification ×10K), (**b`**) (scale bar = 5.0 µm and magnification ×1500), and (**c`**) (scale bar = 5.0 µm and magnification ×1500).

The TEM micrographs revealed that the midgut tissue of creeping bugs in the control group consisted of columnar digestive cells with many secretory vesicles in the apical cytoplasm and a well-defined brush border of short microvilli at the distal border. Microvilli are associated with a well-developed non-cellular peri-microvillar membrane (PMM), which separates the midgut epithelium from its lumen, rather than the peritrophic membrane, as portrayed in Figure 5a. Additionally, a layer of circular muscles was detected beneath the basal lamina of the midgut epithelium. Figure 5b demonstrates that the nucleus of the muscle fiber was oval, with a systematic nuclear envelope. The nuclei of the midgut epithelium materialized in different shapes, with normal chromatin condensation (Figure 5c).

By contrast, the ultrastructure of the polluted midgut showed substantial anomalies in the midgut epithelium, such as nuclear lysis, vacuolization, low cytoplasmic content, and loosening of cells, followed by necrosis and the disintegration of regenerative epithelial cells, as delineated in Figure 5a`–c`. The midgut epithelium’s brush border also underwent alterations, showing a marked reduction in number compared to the control group at the same magnification. Indeed, the microvilli are significantly shorter, misshapen, and may be non-functional. In addition, the microvilli are irregularly thick and distorted (Figure 5a`). Evidently, the basal lamina and the muscle layer were in some parts of the midgut separated from each other. Figure 5b` shows that the circular muscles become morphologically altered, thinner, disintegrated, and sometimes separated from the previous connective tissue layer. In addition, their nuclei emerged indented and irregular in shape. The midgut epithelium from the polluted water showed irregularly shaped nuclei in advanced stages of degeneration, with indented and ruptured nuclear envelopes, lysis of the nuclear content, and cytoplasmic proteolysis. A rupture of the cell membrane and some signs of necrosis in both the nuclei and cytoplasm of the epithelial cells were detectable. Large vacuoles were present in the epithelial layer, and nidi were almost absent (Figure 5c`).

### 3.6. Histopathological and Ultrastructural Examination of Malpighian Tubules

**Figure 6:**
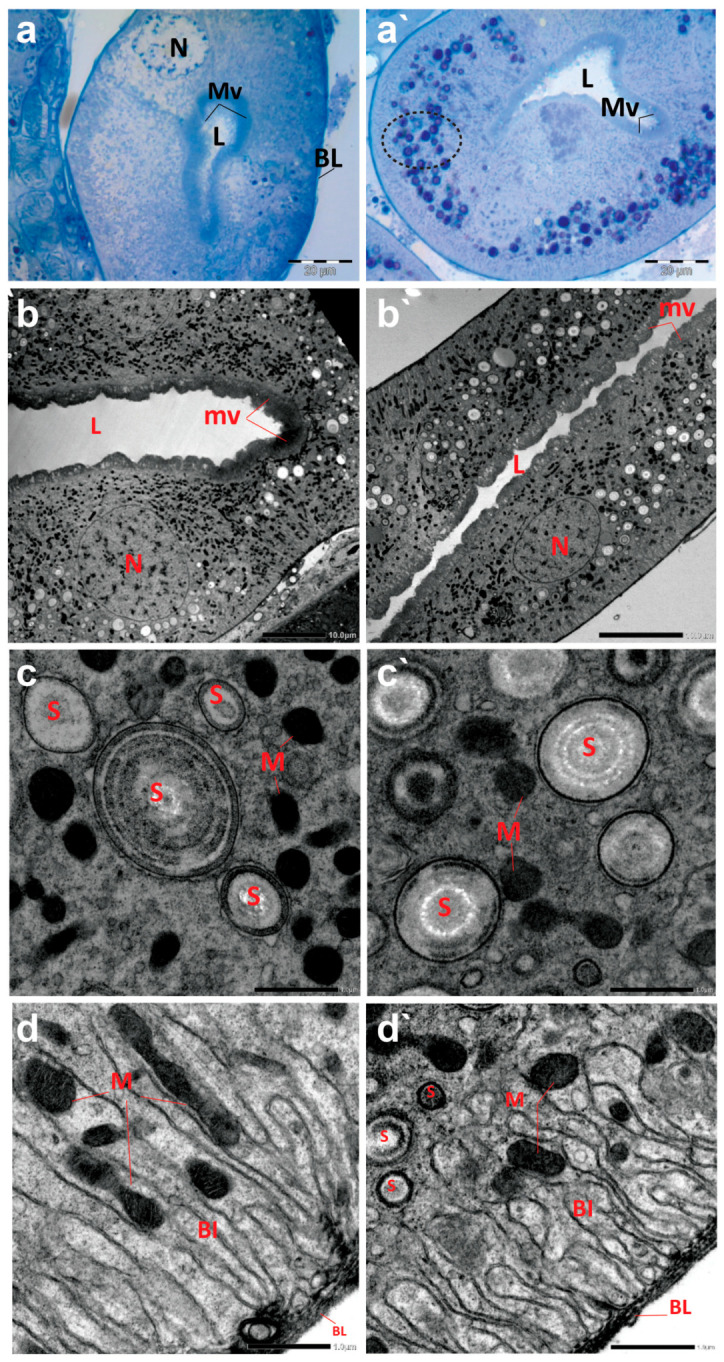
Photomicrographs of semithin sections and transmission electron micrographs of Malpighian tubules (MTs) of creeping water bugs collected from the reference site (A) and the polluted site (B). (**a**) presents histological properties, and (**b**–**d**) exhibit ultrastructure attributes of MTs of creeping bugs from the clean site. (**a**) reveals a semithin cross-section of MTs with a clear cytoplasm, spherical nucleus (N), a lumen (L) lining with microvilli (MV), and basal folds (BL) in the MTs distal part. (**b**) exposes the ultrastructure of MTs, microvilli (mv), lumen (L), and nucleus (N). (**c**) shows spherites (S) and abundant mitochondria (M) in the cytoplasm around them. (**d**) demonstrates the basal labyrinth (Bl) of the epithelial cells of MT. Remarkably, mitochondria (M) between the basal labyrinth (Bl) and the absence of spherites could be discernible. The distal part of the MTs appeared with several basal folds (BL). (**a`**) shows histological abnormalities, and (**c`**,**d`**) illustrate the ultrastructure deformities of MTs of creeping bugs from the polluted site. (**a`**) depicts a semithin cross-section of the MT with numerous cytoplasmic inclusions (encircled), a lumen (L) lining with microvilli (MV). (**b`**) shows the ultrastructure of the MT, manifesting the severe shrinkage in size of MT compared to the control (microvilli (mv), lumen (L), and nucleus (N)). (**c`**) shows a higher number of spherites (S) and a lower number for mitochondria (M), while (**d`**) displays spherites (S) in the basal part of the basal labyrinth (Bl) (mitochondria (M) and basal folds (BL)). (**a**) (scale bar = 20 µm and magnification ×1000), (**b**) (scale bar = 10 µm and magnification ×600), (**c**) (scale bar = 1.0 µm and magnification ×8000), (**d**) (scale bar = 1.0 µm and magnification ×8000), (**a`**) (scale bar = 20 µm and magnification ×1000), (**b`**) (scale bar = 10 µm and magnification ×800), (**c`**) (scale bar = 1.0 µm and magnification ×8000), and (**d`**) (scale bar = 1.0 µm and magnification ×8000).

The cross sections of Malpighian tubules (MTs) of creeping bugs from the control group demonstrated a typical structure, characterized by a single layer of epithelial cells, with a distinct spherical nucleus and a clear cytoplasm, as presented in Figure 6a. In addition, the ultrastructure showed a typical structure of MTs, similar to those previously demonstrated [55]. A long border of microvilli could be discerned opposite the lumen of MTs and at the apical side of the epithelial cells, as illustrated in Figure 6b. Various forms of spherites, surrounded by abundant mitochondria in the cytoplasm, were recognizable (Figure 6c). The distal part of the MTs appeared with several basal folds (BL) and numerous mitochondria in between (Figure 6d). Moreover, the TEM images revealed the absence of spherocrystals in the basal part of the MTs, located between the BL.

In contrast to the control midgut, the histological sections of the MTs from the polluted water bugs revealed many dark blue inclusions inside the cytoplasm of the MTs (Figure 6a`). Compared to the control group, electron micrographs of MTs from the polluted creeping bugs showed several deformities in the form of a critical shrinkage in the size of MTs (Figure 6b`), a fewer number of mitochondria, and a higher number of spherocrystals in the cytoplasm of the epithelial cells (Figure 6c`). In addition, mitochondria were largely absent from the basal labyrinth. Additionally, in contrast to the control sample, numerous spherocrystals of various shapes developed in the basal part of the MTs, suggesting a significant accumulation of metal inclusion bodies within the insect body, as represented in Figure 6d`.

### 3.7. Histopathological and Ultrastructural Analysis of Ovarioles

**Figure 7:**
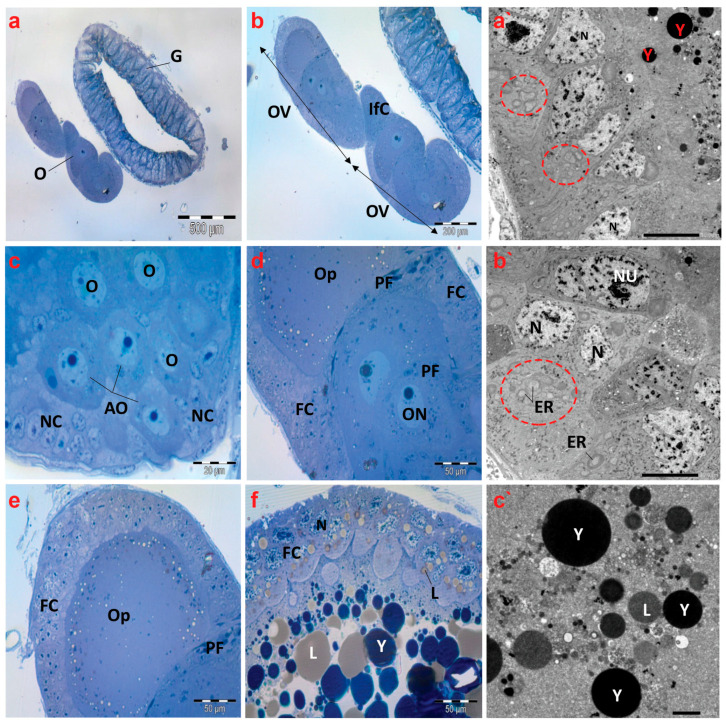
Photomicrographs of semithin sections and transmission electron micrographs of ovarioles of creeping water bugs collected from the reference site (A). (**a**) exhibits a cross-section of the gut (G) and ovary (O). (**b**) indicates a higher magnification of (**a**), showing two ovarioles (OV) and interfollicular cells (Ifc). (**c**) illustrates a cross-section of the tropharium, signifying arrested oocytes (AO), oocytes (O), and nurse cells (NC) or trophocytes. (**d**) presents three previtellogenic oocytes, spindle-shaped prefollicular cells (PF), oocyte nucleus (ON), follicular cells (FC), and ooplasm (OP). (**e**) demonstrates a previtellogenic oocyte surrounded by follicular cells (FC), with ooplasm (OP) and spindle-shaped prefollicular cells (PF). (**f**) shows a vitellogenic oocyte surrounded by follicular cells (FC), with nucleus (N), ooplasm filled with yolk granules (Y) and lipid droplets (L). (**a`**,**b`**) exhibit electron transmission micrographs of ovarioles, manifesting the nucleus (N), binucleated follicular cells (Nu), yolk granules (Y), and numerous endoplasmic reticula (ER) (encircled), while (**c`**) shows yolk granules (Y) and lipid droplets (L). (**a**) (scale bar = 500 µm and magnification ×40), (**b**) (scale bar = 200 µm and magnification ×100), (**c**) (scale bar = 20 µm and magnification ×1000), (**d**) (scale bar = 50 µm and magnification ×400), (**e**) (scale bar = 50 µm and magnification ×400), (**f**) (scale bar = 50 µm and magnification ×400), (**a`**) (scale bar = 10 µm and magnification ×800), (**b`**) (scale bar = 10 µm and magnification ×800), and (**c`**) (scale bar = 2.0 µm and magnification ×2000).

Semithin images of the ovaries of creeping bugs from the unpolluted area depict regular structures defined by telotrophic ovarioles, as shown in Figure 7a,b. The apical region of the ovariole displayed arrested oocytes (AOs), oocytes (Os), nurse cells (NCs), or trophocytes (Figure 7c). Furthermore, the oocytes were observed at different stages of development. Figure 7d,e reveal the first stage, showing the previtellogenic oocytes with spindle-shaped prefollicular cells (PF) between each of them, and a clear ooplasm characterizes previtellogenic oocytes. In the second stage, yolk granules and lipid droplets filled the ooplasm of vitellogenic oocytes (Figure 7f).

Electron micrographs of the control insects’ ovaries revealed regularly occurring binucleated follicular cells and nuclear envelopes that were distinct. Furthermore, the presence of many endoplasmic reticula in the cytoplasm of the follicle cells was noticeable (Figure 7a`,b`). In the ooplasm of a vitellogenic oocyte, normal yolk granules and lipid droplets were also found (Figure 7c`).

**Figure 8:**
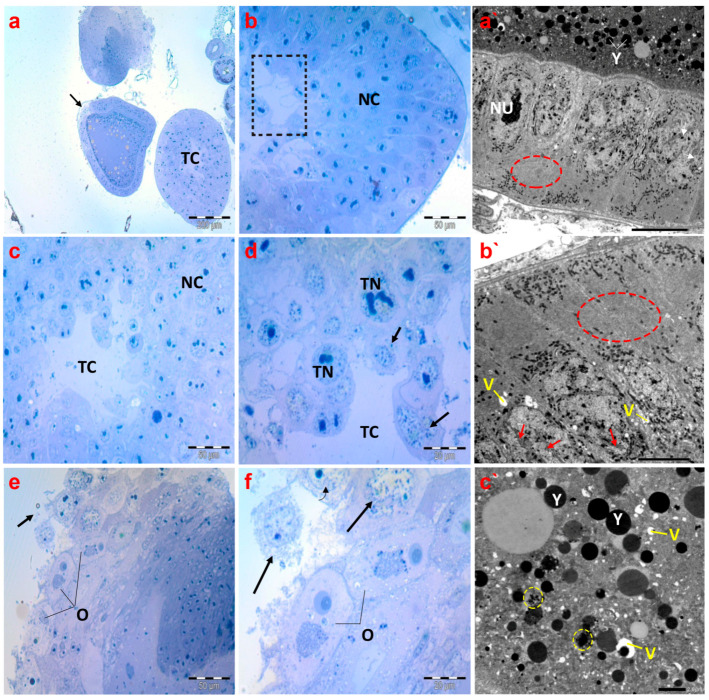
Photomicrographs of semithin sections and transmission electron micrographs of ovarioles of creeping water bugs collected from the polluted site (B). (**a**) presents a cross-section of the tropharium with a centrally located trophic core (TC) and previtellogenic oocyte (arrow). (**b**,**c**) demonstrate cross-sections of the tropharium with a centrally located trophic core (dashed box in (**b**), and TC in (**c**)) and syncytial lobes containing damaged trophocyte nuclei (TN) in (**d**), along with signs of lysis, and nurse cells (NC) with malformations. (**d**) depicts a rupture of the ovariole wall with severe trophocyte lysis (arrows) and damaged trophocyte nuclei (TN) (trophic core (TC)). (**e**,**f**) show the lysis of oocytes (O) also indicated by black arrows. (**a`**,**b`**) represent electron transmission micrographs exhibiting vacuolated cytoplasm (V) and lysis of follicular cell nuclei (arrows). Nucleolus (NU), yolk granules (Y). Crucially, the absence of endoplasmic reticula (encircled) could be perceived. (**c`**) exposes an electron transmission micrograph of a vitellogenic oocyte with vacuolated cytoplasm (V) and yolk granules that are not formed properly (yellow circled), and normal yolk granules (Y). (**a**) (scale bar = 200 µm and magnification ×100), (**b**) (scale bar = 50 µm and magnification ×400), (**c**) (scale bar = 50 µm and magnification ×400), (**d**) (scale bar = 20 µm and magnification ×1000), (**e**) (scale bar = 50 µm and magnification ×400), (**f**) (scale bar = 20 µm and magnification ×1000), (**a`**) (scale bar = 10 µm and magnification ×800), (**b`**) (scale bar = 5.0 µm and magnification ×1500), and (**c`**) (scale bar = 2.0 µm and magnification ×2000).

Concerning the histological inspection of ovaries of creeping bugs collected from the polluted area, the occurrence of critical aberrations in ovarioles is evident. Specifically, the images exhibited no intact ovarioles. Additionally, a cross-section of the tropharium and a malformed previtellogenic oocyte were noticed, as demonstrated in Figure 8a. In addition, we observed nurse cells with evident malformations, including lysis and abnormal chromatin condensation (Figure 8b–d). The rupture of the ovariole wall was the most obvious sign of the damage, in addition to signs of lysis within the oocytes (Figure 8e,f).

Electron micrographs of ovaries of the polluted bugs showed several deformities. The nuclear envelopes of the follicular cells’ nuclei were nearly indistinguishable. In addition to severe nuclear and cytoplasmic lysis, numerous vacuoles were also present. Most notably, the endoplasmic reticula were completely absent from the cytoplasm of follicular cells, as displayed in Figure 8a`,b`. The yolk granules in the ooplasm of the vitellogenic oocytes were either abnormally structured or reduced in size. Moreover, many vacuoles were identified in the ooplasm, along with signs of lysis (Figure 8c`).

## 4. Discussion

Recently, there has been an increasing emphasis on investigating the impact of metals contamination on freshwater ecosystems due to humans’ inevitable exposure [34,56]. Thus, aquatic insects could be utilized as prevalent organisms at the studied site to evaluate the pernicious influence of heavy metals as a consequence of their accumulation in different organs. However, aquatic toxicological studies and metals contamination predominantly focus on the larval stage of insects [57,58,59]. Thus, there is a knowledge gap concerning the impacts of metals exposure on adult aquatic insects, such as *I. cimicoides*, which have not been investigated, to monitor metals toxicity in fresh water in Egypt. In this study, we utilized the adult female *I. cimicoides* to gain a more comprehensive understanding of the deleterious impact of metals contamination in Egypt, represented by the Al Marioteya canal (site B), compared to the adult insects collected from the Al Mansoureya canal, as a clean site (A).

The elemental analysis of water from both sites revealed the incidence of five heavy metals, including Cd, Co, Cr, Ni, and Pb, in addition to the Ca element with, significant increases in their concentrations at site B compared to site A. These findings are remarkably correlated with water analysis parameters, particularly the chemical oxygen demand (COD). It is believed that the COD parameter is a key indicator of water pollution and is broadly applied to assess the equivalent amount of oxygen required for chemically oxidizing the compounds in the water sample [60,61,62]. Therefore, it is employed as a reliable and precise approach to investigate industrial effluents in water for estimating the extent of water contamination and pinpointing the origins of contamination [62]. The incidence of biologically hazardous chemicals, such as heavy metals, phenolic compounds, and pesticides, has a minimal impact on COD assessments, therefore enhancing the precision and reliability of this marker [63,64]. In the present study, COD was remarkably augmented with the increased heavy metals in the Al Marioteya canal (site B), demonstrating a higher level of COD compared to the reference site (site A), which indicates the strong correlation between COD and heavy metals in water samples. These findings are in complete agreement with a previous report, which demonstrated the removal of COD and heavy metals by catalytic ozonation-microbial fuel cell and the *Acidithiobacillus ferrooxidans* leaching process [65].

All heavy metals detected in the polluted water were aggregated in the midgut tissues of *I. cimicoides* from the polluted area, which could be explained by the prevalence of these metals with high concentrations in polluted water in relation to the water from the clean site. Remarkably, the midgut tissues of the polluted insects demonstrated a high concentration of Ca. This is likely due to the exposure of *I. cimicoides* from the polluted site to high levels of heavy metals, causing Ca disorder as a consequence of the expected overabundance of ROS, which triggers oxidative stress [45,66].

It is established that the aggregation of metals within the tissues of various organisms stimulates the productivity of ROS, which in turn instigates a cascade of impairment in different antioxidants and detoxifying enzymes, DNA, and cellular properties [18]. As a result of heavy metals aggregated in the midgut tissues of *I. cimicoides*, biochemical analyses revealed significant augmentation in the levels of MDA, cytochrome P450, and protein carbonyl, associated with the inhibition of SOD, CAT, GPx, and APOX activities in the midgut tissues of the contaminated bugs, compared to creeping bugs from the clean site. In accordance with our findings, previous investigations revealed that the significant rises in MDA and protein carbonyl levels tie in with the overflow of ROS [67,68]. It is believed that MDA is a key biomarker, pointing to the likely malfunctions of diverse molecules, including DNA and proteins, due to their interaction with MDA [40,69,70]. Thus, MDA could be identified as the predominant cause of the genotoxic potential in the midgut tissues of polluted creeping bugs. This explanation is supported by prior investigations, which emphasized the pertinent correlation between DNA impairment and MDA levels in insects and *Wistar* rats [45,71]. Furthermore, lipid peroxidation is well known to incite protein carbonylation [72]. A previous report proposed that the amplification of lipid peroxidation and the protein carbonyl in the tissues of *Nauphoeta cinerea* following exposure to perfluorooctanoic acid allude to the inability of insects to ameliorate the oxidative impairment of both lipids and proteins [72]. Despite the capacity of GPx to detoxify lipid hydroperoxides, a byproduct of lipid peroxidation [44], the polluted bugs showed a lessening in GPx activity. This is completely consistent with the findings of Arafat et al. [44], implying the failure of the GPx in detoxifying lipid hydroperoxides.

In a similar manner, earlier investigations demonstrated decreased SOD and CAT activities as a result of subjection to Cd in the diet of caterpillars of *Lymantria dispar* from the polluted site, in line with our results [73]. Additionally, our previous studies showed that the accumulation of silver nanoparticles at low concentrations in the midgut and ovaries of *Blaps polychresta* led to significant drops in the activity of CAT and SOD [42,45]. Furthermore, the reduction in the APOX level may be related to alterations in the conformational structures of proteins, resulting in inhibition of enzymatic activities, which concurs with previous findings on nickel nanoparticle exposure [74]. These malfunctions imply the development of oxidative stress and impaired antioxidant defense systems in the polluted bugs due to incapacity to modulate the ROS.

Considering the genotoxicity in the midgut tissues of *I. cimicoides*, the comet assay results revealed a substantial proliferation in tail length, tail moment, the percentage of DNA in the comet tail, and the percentage of tailed cells in the polluted aquatic bugs compared to the control bugs. The expansions of DNA impairment and cytochrome P450 match earlier observations by Bernabò et al. [75], who reported a marked increase in all comet parameters, in correlation with an increase in the enzymatic activity of cytochrome P450 in the larvae of *Chironomus riparius* following exposure to Cu. They also stated that the larvae remained viable even with this DNA injury, suggesting the crucial role of cytochrome P450 in metal detoxification. It is assumed that the increased activity of cytochrome P450 in insects plays a pivotal function in the metabolism and detoxification of xenobiotics, which promotes their adaptation to harsh conditions [73]. It could be inferred from these findings that oxidative stress disturbed redox homeostasis and thwarted the potency of the antioxidant system, resulting in DNA injury and protein denaturation [76]. These results correspond to those observed in earlier studies [77].

Concerning the health status of creeping bugs in the polluted site B in our study, a prominent rise in apoptotic and necrotic cells, together with a decrease in viable cells, could be detected. These findings are supported by prior studies, which evidenced the adverse impacts of heavy metals exposure on insect cells by flow cytometry analysis [44,45]. Furthermore, a previous study showed a substantial growth in the apoptotic cells of *Acheta domesticus* exposed to graphene oxide, in comparison to the untreated group [77].

To provide deeper insights into the adverse influence of heavy metals, we also investigate the histological and ultrastructure attributes of the midgut, Malpighian tubules (MTs), and ovarioles of *I. cimicoides* collected from the polluted sites compared to those from clean sites. Accordingly, several anomalies were discernible in the midgut, MTs, and ovarioles of the polluted water bugs. Importantly, the midgut epithelium has been identified as a leading sign of the insect’s intoxication [78].

Consistent with our findings, several previous studies on heavy metal pollution emphasized the histopathological impairments in the midgut epithelium of *Apis millefera* L. [79], *Acheta domesticus* [80], and *Calosoma chlorostictum* [81]. A prior investigation observed ultrastructure deformities in the midgut epithelium of *Drosophila melanogaster* after exposure to cadmium oxide nanoparticles [82]. Moreover, exposure of *A. mellifera* to lead oxide and cadmium oxide nanoparticles, either separately or combined, provoked alterations in the chromatin pattern, the dilation of the endoplasmic reticulum, and swelling of the mitochondria [79]. These reports support the aberrations recognized in the midgut of the polluted creeping bugs in this study.

It has commonly been assumed that the MTs are responsible for the excretion of nitrogenous waste products, in addition to their key role in sustaining osmoregulation and the storage of minerals [55,83]. Of particular interest, spherocrystals, or spherites, are mineral granules with concentric lamellae found in the cytoplasm of MTs that perform a vital function in relation to mineral storage [83,84]. To the best of our knowledge, a few reports probed the histological and ultrastructural impacts of heavy metal-polluted water on insects’ MTs. Sorour et al. [85] reported ultrastructural irregularities in the MTs of *Lethocerus niloticum* obtained from water polluted with heavy metals. In contrast to previous investigations, the MT tissues exhibited no alterations in the cellular structure; however, severe shrinkage in the size of MTs, with a reduction in mitochondrial numbers and an increase in spherocyte numbers, was observed. In line with a previous report [86], we propose that the primary cause of the increase in spherocyte numbers is the heavy metals accumulated in the bodies of the insects from polluted water, which in turn boosted the mineral granules within the MTs.

Based on the histomorphology of adult female creeping bugs in the current study, we found that they have telotrophic meroistic ovarioles, which are similar to those previously studied in Hemiptera, including *Cixius nervosus*, *Javesella pellucida*, *Conomelus anceps* [87], *Gerris lacustris* [88], and *Platymeris rhadamanthus* [89]. In our study, we observed critical impairments in the ovarian follicles of creeping bugs obtained from polluted water. Consistent with our results, earlier investigations showed that the degeneration of the ovarian follicle components was the most obvious damage as a result of solar radiation for *Callosobruchus maculatus* [90]. We suggest that heavy metal pollution in water could explain the reduction in developing oocytes. In the same context, it was noticed that the endoplasmic reticula cisterna was fragmented and dilated in the follicular epithelium of spinosad-treated *Rhynchophorus ferrugineus* [91]. In the present study, we detected indistinct nuclear envelopes within the follicular cells, implying that heavy metal pollution disrupted the oogenesis process in bioindicator insects.

## 5. Conclusions

In conclusion, this study presented the first biomonitoring of heavy metals pollution in the Al Marioteya canal freshwater in Egypt using a creeping water bug (*I. cimicoides*). The water analysis demonstrated the incidence of heavy metals, including Cd, Co, Cr, Ni, and Pb, in addition to the Ca element at high concentrations at this polluted site, associated with a remarkable rise in COD, which is a crucial indicator for water pollution. These heavy metals were accumulated in the midgut tissues of the aquatic insect, resulting in an overflow of ROS, which interfered with the antioxidant defense system and provoked a calcium disorder. Consequently, the deregulation of the SOD, CAT, GPx, APOX, MDA, cytochrome P450, and protein carbonyl levels were reported. The evaluations of DNA impairment and cellular apoptosis are strongly linked with biochemical observations. Moreover, the histopathological and ultrastructural features of the midgut, Malpighian tubules, and ovarioles of *I. cimicoides* were remarkably malformed as a result of exposure to these elements. Overall, the current aquatic bug has demonstrated its potential utility in assessing the accumulation of heavy metals in freshwater and exploring their detrimental effects accordingly. This could help develop efficient approaches to sustaining the quality of freshwater in Egypt.

## Figures and Tables

**Table 1:** Physiochemical parameters of water collected from Al Mansoureya canal (site A) and Al Marioteya canal (site B).

Parameter	Control Site (A)	Polluted Site (B)
pH	7.21	7.27
COD (mg/L)	1580	2400
TOC (mg/L)	3.10	6.80
TDS (mg/L)	1358	1774
DO (mg/L)	7.9	6.8
PO_4_-P (mg/L)	29.1	47.5
NH_4_-N (mg/L)	1.8 ± 0.2	2.1
NO_3_-N (mg/L)	2.0	2.9
NO_2_-N (mg/L)	2.7	6.0
Total N content (mg/L)	157.01	197.88

Chemical oxygen demand (COD), total organic carbons (TOCs), total dissolved salts (TDS), dissolved oxygen (DO).

**Table 2:** Average metal concentrations in freshwater (mg/L) for each study area (mean ± SD), as well as the detection limits (mg/L) of WHO (2021). The *p*-value was determined following the Student’s *t*-test to evaluate the significant difference between elements in water collected from the two sites.

Metals	Site A Conc. (mg/L)	Site B Conc. (mg/L)	*p*-Value	WHO Limits
Ca	2.99 ± 0.79	6.54 ± 0.53	0.003 *	200
Cd	0.008 ± 0.001	0.13 ± 0.01	<0.001 *	0.003
Co	0.01 ± 0.001	0.29 ± 0.03	<0.001 *	—
Cr	0.04 ± 0.007	0.08 ± 0.01	0.007 *	0.05
Ni	0.008 ± 0.001	0.01 ± 0.001	0.005 *	0.07
Pb	0.08 ± 0.01	0.15 ± 0.02	0.02 *	0.01

Asterisks indicate significant differences at * *p* ≤ 0.05.

**Table 3:** Percentage of metals in the midgut of *I. cimicoides* collected from clean site (A) and polluted site (B) based on EDX analysis. The values are presented as mean ± SD, and the *p*-value was determined following the Student’s *t*-test to evaluate the significant difference between the elements accumulated in the midgut tissues of creeping water bugs collected from the two sites.

Metals	Site A	Site B	*p*-Value
Ca	0.27 ± 0.16%	3.93 ± 0.28%	<0.0001 *
Cd	nd	0.28 ± 0.37%	—
Co	nd	0.54 ± 0.31%	—
Cr	nd	0.07 ± 0.04%	—
Ni	nd	0.73 ± 0.45%	—
Pb	nd	0.24 ± 0.22%	—

Asterisks indicate significant differences at * *p* ≤ 0.05, while (nd) points to not detected metals.

## Data Availability

The datasets generated during the current study are available from the corresponding authors upon reasonable request.
